# Alopecia in Belgian Blue crossbred calves: a case series

**DOI:** 10.1186/s12917-019-2140-1

**Published:** 2019-11-15

**Authors:** Matthias Wieland, Sabine Mann, Nicole S. Gollnick, Monir Majzoub-Altweck, Gabriela Knubben-Schweizer, Martin C. Langenmayer

**Affiliations:** 1 Clinic for Ruminants with Ambulatory and Herd Health Services at the Centre for Clinical Veterinary Medicine, Veterinary Faculty, Ludwig-Maximilians-Universität München, Sonnenstrasse 16, 85764 Oberschleissheim, Germany; 2 Present Address: Department of Population Medicine and Diagnostic Sciences, Cornell University, Ithaca, NY 14853 USA; 3 Present Address: German Federal Institute for Risk Assessment, Max-Dohrn-Str. 8-10, 10589 Berlin, Germany; 4 Institute of Veterinary Pathology at the Centre for Clinical Veterinary Medicine, Veterinary Faculty, Ludwig-Maximilians-Universität München, Veterinärstr. 13, 80539 Munich, Germany; 5 Present Address: Institute for Infectious Diseases and Zoonoses, Ludwig-Maximilians-Universität München, Veterinärstr. 13, 80539 Munich, Germany

**Keywords:** Bovine, Crossbred calves, Hair loss, Hypotrichia, Scaling

## Abstract

**Background:**

Alopecia is defined as the partial or complete absence of hair from areas of the body where it normally grows. Alopecia secondary to an infectious disease or parasitic infestation is commonly seen in cattle. It can also have metabolic causes, for example in newborn calves after a disease event such as diarrhoea. In the article, the investigation of a herd problem of acquired alopecia in Belgian Blue (BB) crossbred calves is described.

**Case presentation:**

Several BB crossbred calves had presented with moderate to severe non-pruritic alopecia in a single small herd located in Southern Germany. The referring veterinarian had ruled out infectious causes, including parasitic infection and had supplemented calves with vitamins (vitamins A, B1, B2, B3, B5, B6, B7, B9, B12, C, and K3) orally. Results of the diagnostic workup at the Clinic for Ruminants are presented for three affected calves and findings from a farm visit are discussed. Because of these investigations, an additional four calves were brought to the referral clinic within the first week of life, and before onset of alopecia, in order to study the course of the condition; however, these calves never developed any signs of alopecia during their clinic stay.

**Conclusions:**

Because all other plausible differential diagnoses were ruled out during our investigation, we concluded that the documented alopecia was due to malabsorption of dietary fat and consecutive disruption of lipid metabolism leading to telogen or anagen effluvium. In this particular case, this was caused by a mixing error of milk replacer in conjunction with insufficiently tempered water. We conclude that nutritional, management or environmental factors alone can lead to moderate to severe alopecia in calves in the absence of a prior or concurrent disease event or infectious cause.

## Background

Alopecia is defined as the partial or complete absence of hair from areas of the body where it normally grows. This condition can be caused by abnormality or malfunction of the hair follicles (primary alopecia) or can be associated with inflammation and hypertrophy of the skin and subsequent involvement of the hair follicles (secondary alopecia) [1]. Further, alopecia can be differentiated based on the aetiology: congenital or acquired. Congenital alopecia has been described in different breeds and is caused by genetic defects and oftentimes associated with additional malformations [1]. Acquired alopecia is characterized by a temporary hair loss of different regions of the body and can be caused by bacterial, fungal and parasitic infections, fly infestations (myiasis and warbels) and nutritional deficiencies [2, 3]. Nutrition-related alopecia can be due to malnutrition or malabsorption that lead to caloric deprivation or deficiency of individual components such as proteins, minerals, vitamins and essential fatty acids [2]. Malabsorption of dietary fats is a well-established cause of acquired alopecia in humans [2, 4] and companion animals [5], but its role in the aetiology of acquired alopecia in cattle is less well established. This article describes the investigation of a herd problem of acquired alopecia in Belgian Blue (BB) crossbred calves most likely attributable to a disruption of lipid metabolism due to malabsorption of dietary fat. The investigation included 1) the examination of three animals that were hospitalised after being diagnosed on farm at different stages of the disease, 2) a herd visit to inquire management practices possibly associated with the underlying cause and 3) the study of the clinical course of the disease in four newborn animals that were removed from the farm within the first week of life.

## Case presentation

The Clinic for Ruminants, LMU Munich was contacted by a dairy farmer with a herd problem of hair loss in BB cross-bred calves in December of 2010. According to the owner, calves of both sexes from dairy breed dams [Brown Swiss (BS), Holstein Friesian (HF) and Red Holstein (RH)], sired by different BB bulls through artificial insemination were affected over the course of 5 years. He reported that these calves were born with a normal hair coat. Starting at the age of 2 to 3 weeks, they showed ill thrift, excessive scaling of the neck and head area with areas becoming alopecic shortly after starting at the head and progressing to the dorsal midline, neck and shoulder area. At the age of 8 to 10 weeks, hair started to grow back in the affected areas in all calves. The herd veterinarian began investigating the problem due to the owner’s financial and welfare related concerns. Following physical examinations and samples of affected skin, no apparent cause could be determined by the referring veterinarian in any of the affected calves. Pruritus was absent, no ectoparasites were found and skin scrapings yielded no abnormal results. Skin biopsies obtained by the referring veterinarian were inconclusive in the determination of the cause of alopecia. Treatments of the affected animals with pour-on insecticides [Moxidectin Triclamox Rind Pour-on-Lösung ad us. vet.; moxidectin 0.5 mg/kg body mass (BM), triclabendazole 20 mg/kg BM] and injectable vitamin preparations [dosage/animal: 250,000 IU vitamin A; 25,000 IU vitamin D3; 150 mg vitamin E; 500 mg vitamin C (Ursovit AD3EC, wässrig pro inj.; Serumwerk Bernburg AG, Bernburg, Germany)] did not improve the condition. It was also surprising that purebred dairy calves on the same farm had reportedly never been affected by this disease. After consultation with the herd veterinarian, three animals with typical signs were referred to the clinic for further diagnostic workup and a herd visit was arranged.

According to the owner’s opinion, the three referred male calves, aged 19, 28 and 42 days, presented in various stages of the same condition. They arrived at the clinic over a period of 3 months (January–March 2011). The following management for calf care was identical for all calves: After birth, they were separated from their respective dam and were housed in single box stalls with straw bedding. Over the first 7 to 10 days of life, they received 2 litres of whole milk from their respective dam twice daily. Subsequently, calves were fed two times per day with 4 litres of a commercial milk replacer [Treff Dimilch, Karl Schneider GmbH & Co.KG, Hergatz, Germany (Additional file 1)]. Hay, salt, mineral feed, grain or water were not offered up to this point. Like all other affected calves, the three calves received an oral vitamin mix after the onset of signs as well as a pour-on treatment with an antiparasitic agent (Moxidectin Triclamox Rind Pour-on-Lösung ad us. vet.; moxidectin 0.5 mg/kg BM, triclabendazole 20 mg/kg BM) but hair loss progressed irrespectively.

### Clinical examination at admission, blood sampling procedure and analysis

Immediately upon arrival at the clinic, a clinical examination was performed according to Dirksen et al. [6]. Blood was taken from each animal by puncture of the jugular vein and placed directly into S-Monovette (Sarstedt, Nümbrecht-Rommelsfeld, Germany), anticoagulant (K3 EDTA, 1.6 mg/ml; Sarstedt) and blood gas Monovette (50 IU/ml of calcium-balanced lithium heparin; Sarstedt) tubes. Blood samples were processed immediately and serum was harvested by centrifugation at 3000 rpm for 10 min at 25 °C. Serological parameters, as well as the activity of glutathione peroxidase in whole blood, were determined using an automatic analysing system (Automatic Analyser Hitachi 911; Roche Diagnostics, Indianapolis, IN). Haematological analyses were performed with an automatic haematology analyser (Sysmex F820; Sysmex, Norderstedt, Germany). In addition, the concentration of molybdenum in serum was determined at IDEXX VetMed Labor GmbH, Ludwigsburg, Germany. In two calves (calf 2 and 3), the vitamin C level in serum obtained on the day of hospitalization was determined using liquid chromatography mass spectrometry (MVZ Labor Dr. Limbach, Heidelberg, Germany).

Further, an 8-mm skin biopsy of three different locations (one unaffected, two affected sites) were taken under local anaesthesia, fixed immediately in 10% neutral-buffered formaldehyde and sent to the Institute of Veterinary Pathology, LMU Munich for examination. Formalin-fixed samples were routinely paraffin-embedded and processed for histological examination and stained with haematoxylin and eosin (HE) and Giemsa.

### Clinical signs and clinical pathology

**Fig. 1:**
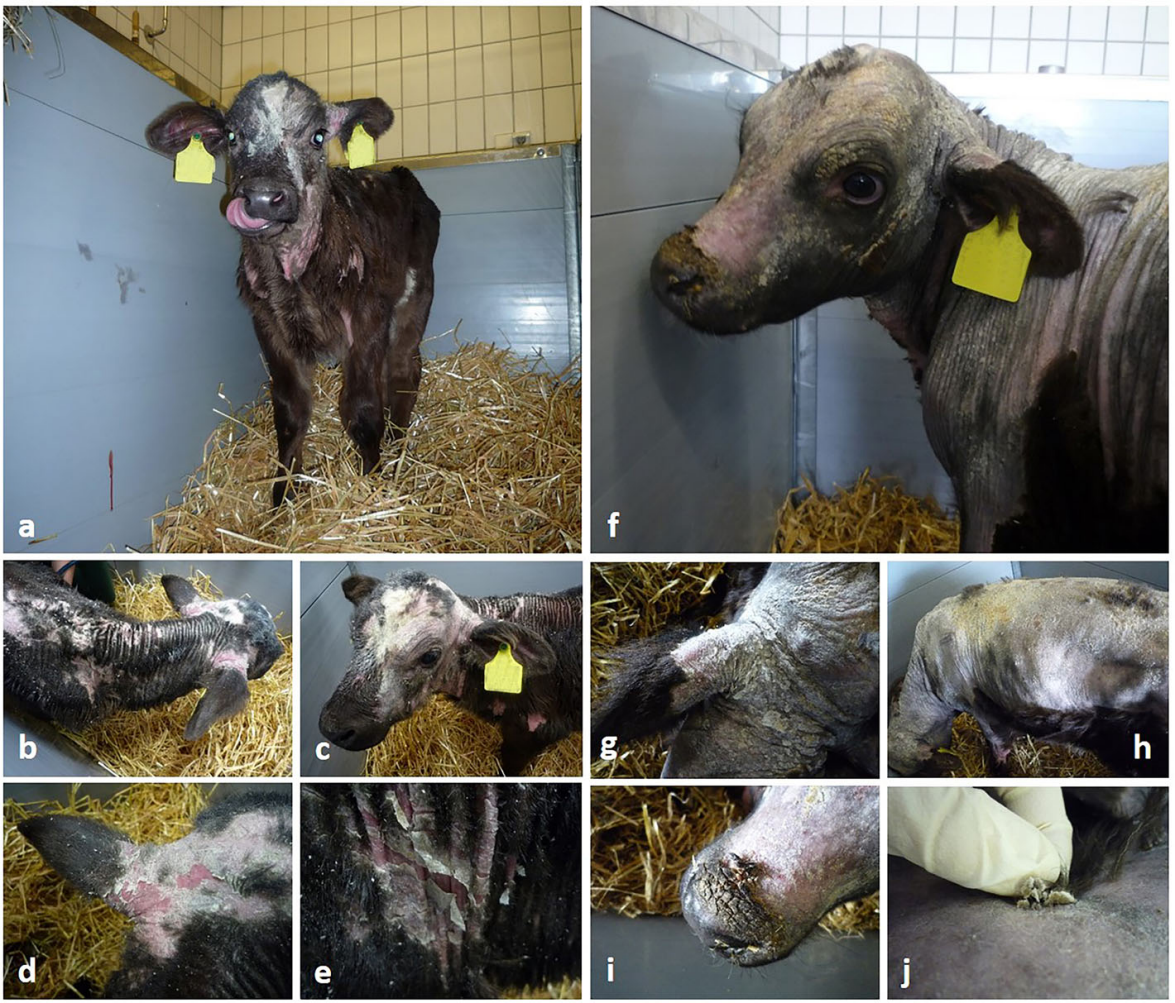
Two herd representatives suffering from alopecia. **a**-**e**: Calf 1; **f**-**j**: Calf 2. Hair loss present on the forehead, around the eyes, the cheeks, along the back and both sides of the neck as well as both elbows. The base of both ears is affected. Excessive scaling with thick, easily removable crusts, most prominent on both sides of the neck

Table 1 depicts baseline characteristics and results of clinical examination of the three calves at the time of hospitalisation. Abnormal clinical findings included the following: abnormal stance with the hind legs gathered underneath the abdomen (calves 1 and 2), whereas calf 3 did not bear weight on the left hind limb. Calves 1 and 2 had cold extremities. A slight reddening of the gingiva around the incisors and a mildly increased pink colour of the mucous membranes were documented in calves 1 and 3. Auscultation of the heart revealed an irregular cardiac arrhythmia with absence of a murmur or jugular vein distension (calves 1 and 2). Hypothermia was detected in two calves (calf 1, 35.9 °C; calf 2, 37.6 °C), whereas calf 3 had an elevated body temperature (40.2 °C). No ulcerations were found on inspection of the oral cavity and interdigital spaces. Hydration status was normal as determined by the evaluation of the skin tent and the position of the eyeballs. In two calves (calves 1 and 2), alopecia was present along the back, on both sides of the neck, on the forehead, around the base of both ears, both cheeks and around the eyes. The reddened skin in these areas was partially covered by thick crusts that could easily be removed. The skin of affected areas was dry and only mildly inflamed; no erosions were found (Fig. 1). By contrast, calf 3 showed only slight scaling on different aspects of the head and the neck.

**Table 1:** Baseline characteristics and clinical findings at the time of hospitalisation of seven Belgian Blue crossbred calves referred to the clinic. Calves 1, 2 and 3 were referred with existing signs of alopecia. Calves 4–7 were picked up at the farm in the first week of life when no clinical signs were apparent. BS, Brown Swiss; BB, Belgian Blue; RH, Red Holstein

Parameter	Calf 1	Calf 2	Calf 3	Calf 4	Calf 5	Calf 6	Calf 7
Cross breed	BS x BB	BS x BB	BS x BB	RH x BB	RH x BB	BS x BB	BS x BB
Age (days)	28	42	19	7	1	2	1
Sex	Male	Male	Female	Female	Female	Male	Male
Body mass (kg)	44.7	57.0	44.2	45.4	45.2	51.5	40.6
Body condition	Poor	Moderate	Moderate	Moderate	Good	Good	Good
Posture	Hind legs gathered underneath abdomen	Hind legs gathered underneath abdomen	No weight bearing on left hind limb	Unremarkable	Unremarkable	Unremarkable	Unremarkable
Behaviour	Unremarkable	Unremarkable	Unremarkable	Unremarkable	Unremarkable	Unremarkable	Unremarkable
Heart rate (beats per minute)	120	100	92	120	112	116	120
Respiratory rate (breaths per minute)	24	40	32	40	36	28	40
Body temperature (°Celsius)	37.6	35.9	40.2	39.1	39.1	39.2	38.2

Haematological and clinical chemistry findings are demonstrated in Table 2. Abnormal findings included polycythaemia (calves 1, 2 and 3), leucocytosis (calves 1 and 3), hyperproteinaemia (calves 1, 2 and 3), hypalbuminaemia (calves 1, 2 and 3), hypocalcaemia (calves 1 and 3), as well as marginal hypokalaemia (calves 1, 2 and 3). Copper concentration and glutathione peroxidase activity were within the respective reference intervals. By contrast, iron (calves 1 and 3) and zinc concentrations (calf 3) were below the respective reference intervals. The vitamin C concentration was within normal range in both tested calves (calf 2, 9.2 mg/L; calf 3, 7.6 mg/L; reference interval Labor Limbach, Heidelberg, 2–20 mg/L).

**Table 2:** Results of haematological analysis and clinical chemistry at the time of hospitalisation of seven Belgian Blue crossbred calves referred to the clinic. Calves 1, 2 and 3 were referred with existing signs of alopecia. Calves 4–7 were picked up at the farm in the first week of life and moved to the clinic before signs appeared. Reference intervals for German Simmental calves, established at the Clinic for Ruminants, LMU Munich, Germany unless otherwise stated. Values above the reference interval are marked with ↑, and those below the reference interval with ↓

Parameter	Unit	Reference interval	Calf 1	Calf 2	Calf 3	Calf 4	Calf 5	Calf 6	Calf 7
Red blood cells	× 10^12^/L	5–8	12.8 ↑	13.4 ↑	9.3↑	8.7↑	9.1↑	11.0↑	9.3↑
Haemoglobin	mmol/L	6.2–8.7	8.9↑	9.4↑	7.3	7.2	7.3	9.6↑	7.6
Haematocrit	%	30–36	52.6↑	62.2↑	40.2	39.1	41.1	47↑	38.7
Mean Corpuscular Volume (MCV)	fl	40–60	41.1	37.4↓	43.3	45.2	45.3	42	41.6
Mean Corpuscular Haemoglobin Concentration (MCHC)	mmol/L	16–21	17.9	18.4	18.2	18.4	17.8	21.0	20.7
Mean Corpuscular Haemoglobin (MCH)	fmol	0.9–1.4	0.7↓	0.7↓	0.8↓	0.8↓	0.8↓	0.9	0.9
Platelets	× 10^9^	200–800	718	662	754	754	368	339	529
White Blood Cells (WBC)	× 10^9^	4–10	25.0↑	8.5	23.2↑	5.3	10.3↑	12.7↑	17.2↑
Urea	mmol/L	< 5.5	5.9↑	5.0	3.9	19.6↑	2.3	3.2	2.6
Creatinine	μmol/L	< 110	43.2	42.4	92.5	248.4↑	131↑	153.2↑	85.5
Urea/Creatinine		30–50	136↑	118↑	42	79↑	18	21	30
Total Protein (TP)	g/L	55–70	51.1↓	53.4	49.6↓	48.3↓	58.7	51.5↓	51↓
Albumin	g/L	30–40	27.4↓	26.2↓	21.5↓	23.7↓	17.5↓	28.2↓	24.5↓
Sodium	mmol/L	135–150	139.3	127↓	130↓	131.5↓	134.1↓	133.7↓	132↓
Potassium	mmol/L	4–5	3.9↓	3.6↓	4.5	4.3	4.5	5.1	4.4
Calcium	mmol/L	2–3	1.2↓	1.2↓	2.1	1.1↓	1.3↓	1.2↓	1.2↓
Phosphorus	mmol/L	1.5–2.1	2.3↑	1.8	1.7	2.6↑	2.3↑	2.1	2.3↑
Iron	μmol/L	12–44	9.7↓	23.2	8.9↓	24.8	25.9	13.7	16
Copper	μmol/L	8–39	8.2	9.0	18.1	11.2	4.3↓	7.6↓	10.8
Zinc	μmol/L	10–20	10.9	17.3	7.7↓	8.6↓	12.3	13.0	15.4
Molybdenum	μg/L	< 10^a^	< 10	< 10	< 10	< 10	245	99	294
Glutathione peroxidase	U/gHb	> 250	310	396	352	440	308	442	344
Vitamin C	mg/L	2-20^b^	n.a.	9.2	7.6	10	7	9.8	4.1

^a^Reference interval established at IDEXX VetMed Labor GmbH, Ludwigsburg, Germany

^b^Reference interval established at Labor Limbach, Heidelberg, Germany

### Histological findings

**Fig. 2:**
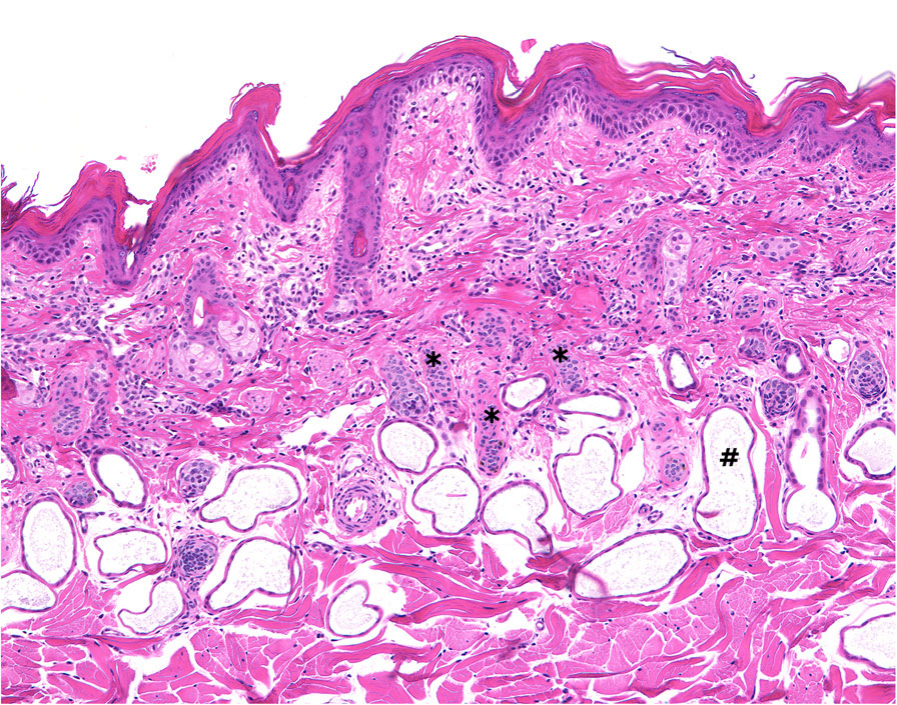
Calf 1 skin histology at day of presentation: Superficial laminar orthokeratotic hyperkeratosis corresponding to the clinical picture (flakes). Hair follicles are diffusely reduced in size (asterisks). Note: Normal apocrine glandular dilation (#) of bovine skin

In samples of affected skin, there was lamellar orthokeratotic hyperkeratosis of the epidermis with keratin flakes and few superficial crusts. In the dermis, hair follicles were small and follicular lumina contained only few hair shafts. Further, minimal perivascular superficial lymphocytic infiltration/inflammation was documented. There was no evidence of relevant bacterial or fungal infection, parasitic infestation or autoimmune disorder (Fig. 2).

### Treatment and clinical course

Two calves (calves 1 and 2) received no treatment throughout the duration of the hospitalisation. They were offered 3 litres of a commercial milk replacer twice daily and received hay and calf starter (grain) free choice. Both calves drank well and started to eat with good appetite over the following days. New growth of hair and a reduction of scaling were noted starting at the age of 7 weeks in both calves (1 and 3 weeks after arrival at the clinic, respectively). The initially thin hair had fully grown back by the time of discharge at the age of 14 (calf 2) and 18 (calf 1) weeks. At this time, the regrown dark hair coat could be easily differentiated from the slightly lighter original, intact hair coat. A control visit 9 months after discharge showed a normal hair coat and episodes of hair loss had not been observed.

**Fig. 3:**
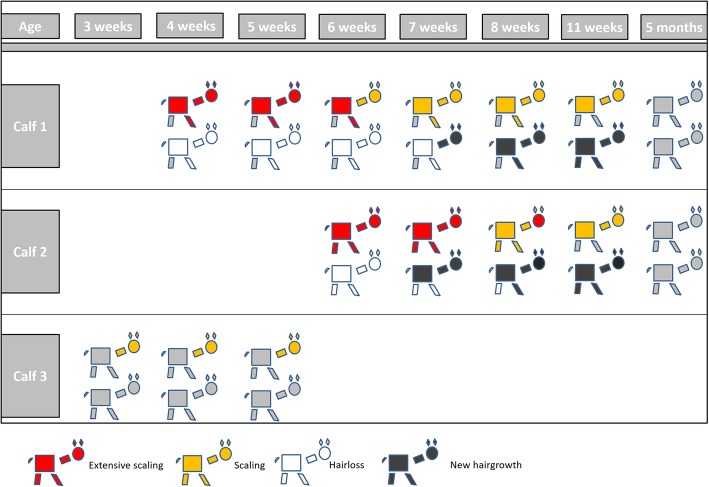
Clinical course of alterations of skin and hair coat in different body regions of three Belgian Blue crossbred calves referred to the clinic over a period of 3 months. The first row indicates alterations of the skin (i.e., scaling); the second row depicts presence or absence of hair loss and new hair growth

Calf 3 was diagnosed with a septic arthritis of the left tarsal joint. Initial treatment consisted of cefquinome (1 mg/kg BM; s.c.; Cobactan 2.5% ad us. vet.; MSD Animal Health Innovation GmbH, Schwabenheim, Germany) and meloxicam (0.5 mg/kg BM; s. c.; Metacam 20 mg/ml ad us. vet.; Boehringer Ingelheim GmbH, Ingelheim, Germany). Five days after admission, an arthrotomy was performed. After a temporary improvement, the lameness and the general condition of the animal deteriorated and the animal was euthanized 12 days after the surgical intervention. Only scaling in the head and neck area had been observed up to this point. Figure 3 depicts the clinical course of alteration of skin and hair coat of the three calves.

### Herd investigation

After consultation with the dairy farmer and the herd veterinarian, a herd visit was arranged. The farm was located in Southern Germany in the vicinity of two other farms on top of a hill (~ 800 m above sea level). At the time of the visit, the herd consisted of 27 cows (20 BS, 3 RH, 3 HF, 1 BS x HF), five heifers (BS) and seven calves. The rolling herd average for the previous year was 6551 kg/cow/year. All adult animals were housed in the same tie-stall barn with mattresses and straw bedding.

### Feeding and management

The ration for the lactating animals consisted of grass silage, hay and two different concentrate feeds [Bovigold 164, RKW Süd, Regensburg, Germany (Additional file 1); custom-made corn pellets] according to the estimated current milk yield (one or several scoops full). Chemical analysis of the grass silage, hay and corn pellets was carried out at the Institute of Physiology, Physiological Chemistry and Animal Nutrition (LMU Munich). Results per kg dry matter are listed in Additional file 2 and an excerpt of the computer-assisted calculation of the lactating cow ration is displayed in Additional file 3. Because the owner had no access to a scale, the ration could only be estimated and was determined to be 20 kg of grass silage and 3 kg of hay (wet weights). For a cow in the peak of lactation, the owner assessed the amount of concentrate fed to be about 5 kg (3 kg grain mix, 2 kg pellets). Because feeding of mineral mix was regarded sporadic at best, it was not included in the calculation. The estimated ration contained 22% raw fibre (14% structured) and 10% of crude protein. An excess supply of fibre (grass silage with very high dry matter content) and a lack of protein (negative ruminal nitrogen balance) became apparent. According to model estimations, a cow in the peak of lactation received enough feed to produce 23.2 kg of milk.

Dry cows and heifers received only grass silage and hay. Mineral feed [Fulminant MV/Fulminant Phos, Fulminant GmbH, Stockach-Zizenhausen, Germany (Additional file 1)] was given sporadically (every 4–7 days) to the lactating animals and sometimes also to the dry ones. All cows had access to pasture during the summer months. All farms in the vicinity received water from the same well. Hay and grass silage were produced on the farm. Manure was spread on all pastures; no other fertilizer had been used during the last 10 years. Salt was not offered as part of the ration.

Calves were born in the tie-stall area. After removal from the dam, they were either housed in individual or shared box stalls. Each calf received colostrum and milk from its respective dam for the first 7 to 10 days of life when they were switched to a commercial milk replacer [Milkibeef Top, Trouw Nutrition Deutschland GmbH, Burgheim, Germany (Additional file 1)]. During the last months before the investigation, the milk replacer was changed to a different brand [Treff Dimilch, Karl Schneider GmbH & Co.KG, Hergatz, Germany (Additional file 1)] but the problem persisted. No standard operating procedure on how to mix the milk replacer, specifying amount, mixing and feeding temperature was available. Upon request, the owner stated that he estimated the amount of milk replacer and that mixing temperature ranged between cold and hand warm varying dependent on the availability of warm water in the barn. The owner stated that hair loss had occurred in calves fed whole milk only, but no records were available to review feeding management for affected calves. For some months, BB crossbred calves had also received three 10 ml doses of an oral vitamin mix for the first 3 days of life [Supervitamine, BEWITAL petfood GmbH & Co.KG, Südlohn, Germany (Additional file 1)]. At the age of 6 weeks, calves were offered free choice hay, grain, and water. Calves were weaned around the age of 3 months.

### Examination of pre-weaned calves

**Fig. 4:**
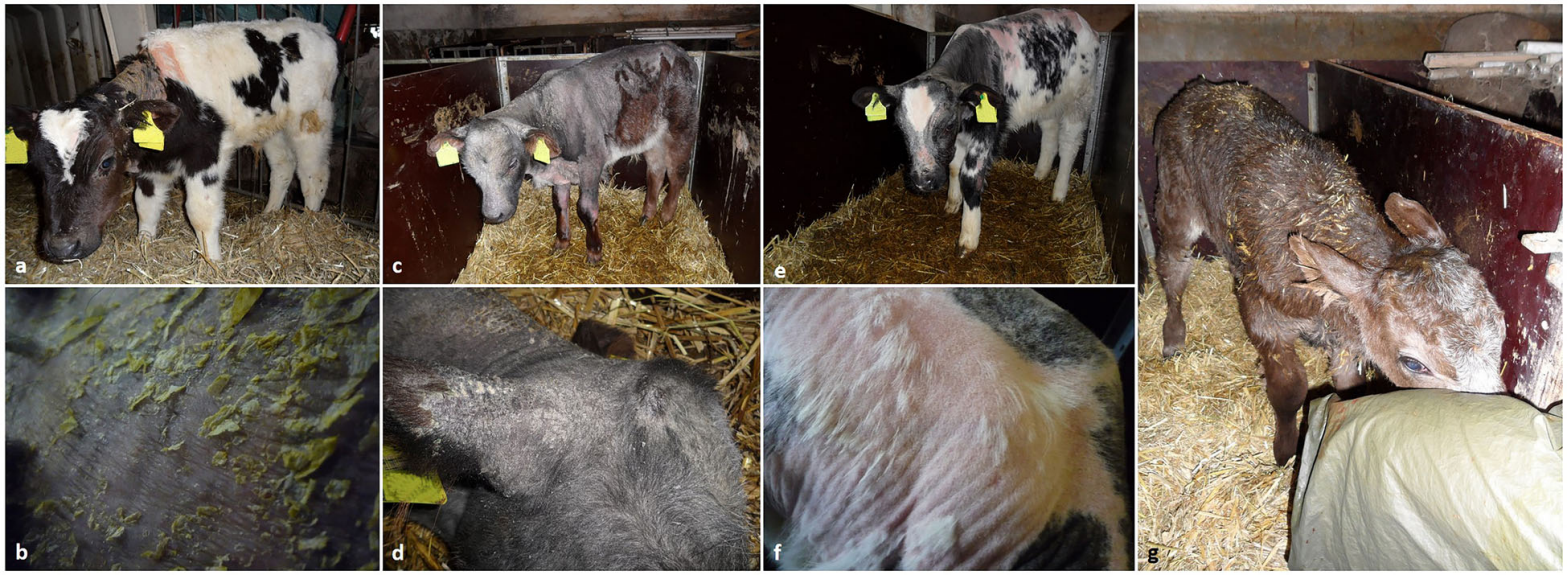
Four Belgian Blue crossbred calves on the farm housed in box stalls. Images taken during the herd visit. **a** and **b**: BB x HF crossbred calf, 6 weeks old, with extensive hair loss around the neck, withers and around the eyes. **b**: extensive scaling of the skin at the neck. **c** and **d**: Nine-week-old BB x BS crossbred calf with history of extensive alopecia and fine growth of hair, note the posture with hind legs gathered underneath the abdomen. **d**: Head and ear base showing slight scaling and fine growth of hair. **e** and **f**: BB x HF crossbred calf, 9 weeks old, with history of alopecia and fine growth of hair. **f**: Withers and shoulder area showing fine growth of hair. **g**: Newborn BB x BS crossbred calf with intact hair coat

Seven calves were examined at the time of the herd visit. Four younger calves (three BS, one BB x BS) between 1 and 10 days of age, as well as three older calves (BB x HF, BB x RH, BB x BS) aged 6 to 9 weeks. All crossbred calves were male; the three female purebred BS calves were intended to be replacement heifers. The younger calves showed no abnormalities on physical exam except for one calf suffering from neonatal diarrhoea and fever; no abnormalities of the skin and coat were detectable. The three older calves showed hair loss around the head, neck, elbows, shoulders and back (Fig. 4). In all three calves, alopecia and scaling had started around the age of 3 weeks and hair started to grow back at the age of approximately 6 weeks. All older calves were poorly developed and showed low body condition compared with BB calves of the same age. Further findings included an irregular arrhythmia on auscultation of the heart in a nine-week-old crossbred calf. Skin tent and position of the eyeballs revealed no clinically detectable signs of dehydration. Blood samples were taken from all calves as described above. All four BB crossbred calves had elevated values for haematocrit (51–59%; mean, 54%; reference interval Clinic for Ruminants, LMU Munich, 30–36%) and erythrocyte counts (12.5–14.6 ×  10^12^/L; mean, 13.50 × 10^12^/L; reference interval Clinic for Ruminants, LMU Munich, 5–8 × 10^12^/L). Levels of albumin and total protein were not indicative of dehydration in these calves [7]. Haematological and biochemistry parameters as well as trace mineral levels and glutathione peroxidase activity were unremarkable with the exception of reduced concentration of total protein in a two-day-old calf had (42.40 g/L; reference interval Clinic for Ruminants, LMU Munich, 55–70 g/L), indicating failure of transfer of passive immunity.

### Examination of adult animals

Rumen fill was good to very good in almost all adult animals. Fourteen out of 27 adult animals showed claw deformations due to overgrowth and lack of claw trimming and four out of these 14 exhibited signs of lameness or decubital sores of the extremities. Body condition score (BCS) was determined for all adult animals according to Edmonson et al. [8]. Four animals in different stages of lactation had a BCS of ≤2.5/5.

Blood samples taken from six recently fresh cows [1–42 days in milk (DIM)] were analysed and results of haematology and blood chemistry showed no abnormalities. Concentration of beta-hydroxybutyrate were between 0.5 and 0.9 mmol/L for these animals. Six urine samples of lactating animals were tested and results were unremarkable apart from four samples with low sodium concentrations (13.0–16.0 mmol/L; reference limit Clinic for Ruminants, LMU Munich, > 20 mmol/L).

### Further investigations

After consultation with the owner and the herd veterinarian, another four BB crossbred calves between 1 to 8 days of life were brought to the clinic to study the clinical course of the disease from the beginning on. All calves had received colostrum from their respective dams and received whole milk before they were picked up. In order to reproduce the situation on the farm, all four calves received the same commercial milk replacer twice daily. Water, hay and calf starter (grain) were offered ad libitum. They received no further treatments. All calves were examined clinically upon arrival and blood samples were obtained to analyse as described above, including determination of vitamin C content in serum. The presence or absence of hair loss was documented daily. Baseline characteristics and results of the clinical examination are presented in Table 1. Abnormal findings were limited to an irregular cardiac arrhythmia in three calves (calves 4, 5 and 6). Table 2 depicts results of haematology and clinical chemistry including vitamin C level in serum. None of the four calves developed the typical lesions including scaling and hair loss while hospitalized in the clinic during the following 3 months.

## Discussion and conclusions

Alopecia in young ruminants is rare and in the experience of the authors usually affects calves during or after an episode of severe diarrhoea or ruminal drinking. In a study of Lorenz et al. [9] the authors concluded that the hair loss after longer periods of disease might be due to either the formation of potentially toxic substances (such as D-lactate) or to deficiency of essential substances culminating in the massive simultaneous defluxion of hair at different stages of the hair cycle. Alopecia in calves has also been reported due to genetic disease [10, 11], fungal infections and parasite infestation [12], trace element [13] or vitamin deficiencies [3] and after the feeding of certain milk replacers using plant-sourced fats [14].

Because the dams of the affected calves were of different breeds (BS, HF, RH) and because at least two different BB bulls had been used, the possibility of a genetic defect was placed low on our list of possible causes. A very similar skin condition to the one described exists as an autosomal recessive hereditary form known as congenital progressive alopecia, but occurs concurrently with anaemia in Polled Hereford calves [10, 15, 16]. However, this disease is progressive in nature and affects calves of the same sire [17].

Because skin biopsies and scrapings showed no indication of a fungal, bacterial or parasitic infection and because pruritus was absent, we ruled these out as possible aetiologies. Moreover, topical treatments with avermectines by the referring veterinarian had not improved or prevented hair loss and hair loss was self-limiting once calves were weaned.

Although liver biopsies are considered the gold standard for the monitoring of trace element status, we had no indication that such an invasive procedure was justified. Therefore, we relied on the results of serum samples that were inconclusive and did not point us into the direction of a lack of a certain trace element.

Our data regarding vitamin supply were incomplete because we did not have values for the vitamin contents of whole milk, but only for the two milk replacers. Hair loss in calves similar to this condition was described by Blowey and Weaver [3] as idiopathic alopecia attributed to milk allergy or vitamin E deficiency. Bouvet et al. [18] described a case of a 3-week old Charolais calf with progressive hair loss and attributed it to folic acid deficiency. Omission of mineral and vitamin balancer from a commercial milk replacer has produced a similar clinical picture in newborn lambs [19]. A number of facts led us to believe that vitamin deficiency could not be the underlying problem. First, two different milk replacers enriched with different levels of vitamins, including vitamin E, were fed. Furthermore, after the owner had become aware of the ongoing problem, he administered a supplement enriched with vitamin E and folic acid to the calves, which did not change the course of the disease. Additionally, errors in milk replacer composition and omission of certain ingredients such as minerals or vitamins appears unlikely since both brands are commonly fed to calves in Germany and the problem was ongoing for 5 years in which different lots of both replacers would have been fed.

Vitamin C deficiency has also been reported as a cause of hair loss in growing calves with nonpruritic seborrhoea, crusting, alopecia, easy hair epilation starting on the head and limbs [5, 20]. Although the mechanism for this disease complex is unclear, it is unlikely the cause in this herd problem because levels of vitamin C in serum were well within the reference interval in the two calves tested during the active period of alopecia, as well as the four hospitalized newborn calves.

Because overall management on the farm showed deficiencies, a recent change in use of different milk replacers had happened and due to the rather unreliable feeding strategies described by the owner, we assume that information about feeding of the calves and cows was incomplete. This possibility is supported by the fact that BB crossbred calves that were brought to the clinic shortly after birth never developed the same signs as the crossbred calves raised on the farm. Therefore, we assume that the aetiology was associated with on-farm management. Although the owner reported feeding a certain amount of either whole milk or milk replacer at a certain concentration on a regular basis, the absence of a standard operating procedure, a weigh scale, mixing equipment (e.g., wire whisk) and thermometer suggested substantial deficits in the on-farm calf-feeding program. This is further supported by the fact that calves examined in the clinic were underweight and poorly developed as were the older calves on the farm. Among the aforementioned factors, mixing and feeding temperature most likely are of particular significance when trying to explain the aetiology of the observed phenomenon. Incorrect mixing temperature often results in reduction of overall solubility of milk replacer, impact fat emulsification and adversely affect ingredient digestibility. This may have led to a subsequent metabolic lipid disorder. Indeed, feeding of milk replacers containing certain fatty acids and high amounts of fat has been described as a cause of alopecia [14]. Together with the possibility of an incompletely emulsified milk replacer/water mixture, this appears as the most likely causative factor of the on-farm problem. As outlined by Gründer and Musche [21], the absorption of insufficiently decomposed, unphysiological fatty acids of vegetable origin, particularly when mixed with insufficiently hot water, may lead to excretion of unphysiological fatty acids via the sebaceous glands. This can impact the hair growth cycle, resulting in telogen or anagen effluvium. A second possible result of the mixing error and potential explanation for the documented alopecia could have been a subsequent decrease in the availability of essential fatty acids (i.e., linoleic acid and alpha-linolenic acid). Several researchers reported similar lesions in lambs and goat kids [22] and calves [23] following experimentally induced deficiency of polyunsaturated fatty acids. However, because concentration of polyunsaturated fatty acids were not determined in affected calves, this possible explanation remains speculative.

Especially calves of fast growing breeds with high metabolic rates, such as the BB calves, might be susceptible to such a disturbance in lipid metabolism. This might also explain why only crossbred calves were affected while purebred BS, HF and RH calves were not. Another explanation could have been preferential feeding of whole milk to replacement heifers whereas bull calves could have been preferentially fed with milk replacer. The fact that hair regrowth started a few weeks after hay, grain and water was offered might be due to the associated ruminal development. This coincides with a change in nutrient availability and digestion [24] and may further support our theory of disruption of lipid metabolism in the pre-weaned stage.

A recommendation of feeding an amount of at least 15% of each calf’s body weight as whole milk or milk replacer (following the mixing instructions supplied by the manufacturer) was made and we recommended that hay and water be offered from the first days of life. In addition, the owner was advised to offer a commercial calf starter containing trace elements to all calves starting in the second week of life.

Although the haematocrit can be above the adult cattle reference interval in calves [16], the values for haematocrit and erythrocyte count were clearly above the two cited reference intervals for calves. The cause for the polycythaemia found in all affected animals and cardiac arrhythmia in six animals could not be determined thus far. In ruminants, polycythaemia is usually diagnosed in cases of dehydration, which was ruled out in all cases by clinical examination (lack of prolonged skin tent, normal position of the eye) and laboratory analysis (physiological concentrations of total protein and albumin). Other causes such as systemic hypoxia due to high altitude, chronic pulmonary disease, cardiac shunt, renal tumours or myeloproliferative disorders [7] were deemed to be extremely unlikely based on the history and laboratory results. In humans, cardiac arrhythmia has been associated with dyslipidaemia and elevated plasma cholesterol [25–27]. In calves, hypercholesteraemia has been documented in conjunction with feeding different milk replacers that contained fatty acids from different animal and plant sources [21]. Although this relationship remains speculative in the absence of information regarding fatty acids concentration and is only attributable to calves that received milk replacer (calves 1 and 2), this possible association should be considered and tested in future cases of alopecia in pre-weaned calves.

Feeding of the cows was considered inadequate and the lack of nutrient supply was reflected in the low herd productivity. The herd performance of 6551 kg per 305-day lactation period is below the German average for Brown Swiss cows of over 7000 kg and well below the genetically possible yearly yield of 8000 to 9000 kg [28]. Cows should not drop under a BCS of 2.5 at any time as was the case in this herd indicating weight loss due to lack of nutrients, chronic disease or both [29]. Because of these facts and the data obtained from monthly productivity reports (LKV Bayern, data not presented), the owner was advised to consult with a dairy nutritionist regarding his feeding strategy. In addition, the owner was advised to schedule a routine herd visit with a local foot trimmer as soon as possible and to continue routine foot trimmings thereafter. The sodium deficiency (urinary sodium excretion under the reference limit in four out of six samples) was communicated to the owner and it was recommended to offer salt lick blocks to all animals.

The authors are aware that in this particular case management data were incomplete, and possibly inaccurately reported in parts by the owner and it is possible that certain facts were concealed during this herd health investigation (such as true frequency, regularity and amount of feed and milk offered, topical treatments that might have been irritating to the skin, etc.). Yet, by failing to replicate the disease process off farm, we infer that nutritional or management factors alone led to the observed moderate to severe alopecia in calves in the absence of a prior or concurrent disease event.

Because all other plausible differential diagnoses were ruled out, we conclude that the documented alopecia has been due to malabsorption of dietary fat in accordance with previous reports [1, 21]. In this particular case, this was likely caused by a mixing error of milk replacer in conjunction with insufficiently heated water. We attributed the disruption of the hair growth cycle resulting in telogen or anagen effluvium to a subsequent lipid metabolic disorder. We demonstrated this by failure to replicate a similar condition in calves that were moved off the farm within a week of birth. Practitioners facing a similar situation should be aware of this possible aetiology when investigating a herd outbreak of alopecia, especially when other apparent and common causes of hair loss are ruled out and should review milk replacer feeding practices in detail.

## Supplementary information

**Additional file 1.** Feed components contents. Composition and contents of milk replacers, vitamin supplement, mineral feeds and grain mix that were fed/applied to calves and cows at the dairy farm.

**Additional file 2.** Results of feedstuff analysis. Percent dry matter, net energy content for lactation, percent protein, percent fibre, percent fat, percent ash, calcium, phosphorus, sodium, potassium, magnesium, iron, zinc, copper, chloride and manganese of grass silage, hay and corn pellets fed to cows at the dairy farm.

**Additional file 3.** Summary of the computer assisted calculation of the lactating cow ration. Calculated wet weight, dry matter, net energy content for lactation, raw protein, ruminal nitrogen balance, calcium, phosphorus, magnesium and sodium content of the lactating cow ration using a computer assisted calculation program.

## Data Availability

The datasets used and analysed during the current study are available from the corresponding author on reasonable request.

## Abbreviations

BB: Belgian Blue
BM: Body mass
BS: Brown Swiss
Ca: Calcium
GSH-Px: Glutathione peroxidase
HF: Holstein Friesian
Mg: Magnesium
Na: Sodium
NEL: Net energy of lactation
P: Phosphorus
RH: Red Holstein
RNB: Ruminal N-balance
