# Supplementary material for: Alopecia in Belgian Blue crossbred calves: a case series
Source: BMC Vet Res. 2019 Nov 15;15:411. doi: 10.1186/s12917-019-2140-1 (PMC6858713; doi:10.1186/s12917-019-2140-1)
Supplement: Supplementary file 1 — Additional file 1. Feed components contents. Composition and contents of milk replacers, vitamin supplement, mineral feeds and grain mix that were fed/applied to calves and cows at the dairy farm. [file 12917_2019_2140_MOESM1_ESM.docx]

**Additional file 1 – Feed components contents**

*Milkibeef Top (milk replacer old), Trouw Nutrition Deutschland GmbH, Burgheim, Germany*

Composition:

40.0% skimmed milk powder, 26.0% whey powder, 17.0 vegetable oil (palm oil, coconut oil, sunflower oil), 9.0% whey powder, partially sugar extracted, 3.0% wheat flour, 2.0% gluten

Contents:

22.0% crude protein, 1.7% lysine, 0.1% crude fibre, 7.5% ash, 1.0% calcium, 0.8% phosphor, 0.5% sodium

Additives per kilogram:

40,000 IU Vitamin A, 4,000 IU Vitamin D (Vitamin D3), 200 mg Vitamin E (alpha-Tocopherol acetate), 200 mg Vitamin C, 200 mg iron (Fe-(II)-carbonate), 9 mg copper (Cu-(II)-sulphate, 120 mg zinc (zinc sulphate), 30 mg manganese, 4 mg iodine, 0.45 mg selenium (sodium selenite), 109 CFU Enterococcus faecium (E 1708)

*Treff Dimilch (milk replacer new), Karl Schneider GmbH & Co.KG, Hergatz, Germany*

Composition:

57.5% skimmed milk powder, 18.0% vegetable oil, 9.5% whey powder, 5.5% soy protein, 4.5% wheat starch, 3.5% dextrose

Contents:

22.0% crude protein, 20.0% raw fat, 1.95% lysine, 0.15% raw fibre, 7.1% ash, 1.0% calcium, 0.7% phosphor, 0.35% sodium

Additives per kilogram:

75,000 IU Vitamin A, 7,500 IU Vitamin D (Vitamin D3), 115 mg Vitamin E (alpha-Tocopherol acetate), 120 mg iron (Fe-(II)-carbonate), 5 mg copper (Cu-(II)-sulphate, 50 mg zinc (zinc sulphate), 40 mg manganese, 0.1 mg iodine, 0.25 mg selenium (sodium selenite), 0.15 mg cobalt

*Supervitamine (vitamin supplement calves), BEWITAL petfood GmbH & Co.KG, Südlohn,* Germany

Additives per Litre:

20,000,000 IU Vitamin A, 200,000 IU Vitamin D3, 30,000 IU Vitamin E, 500 mg Vitamin B1, 1,250 mg Vitamin B2, 2,000 mg Vitamin B6, 20 mg Vitamin B12, 20,000 mg Vitamin C, 3,000 mg Vitamin K3, 20,000 mg nicotinic acid, 6,500 mg pantothenic acid, 50 mg folic acid, 10 mg biotin, 1,000 mg inosite

Recommended dose: 10 ml/calf for a total of three days

*Fulminant MV Premium (mineral feed, lactating animals), Fulminant GmbH, Stockach-Zizenhausen, Germany*

Composition:

Calcium phosphate, sodium bicarbonate, calcium carbonate, magnesium oxide, sodium chloride, grape seed extract*, sugar beet molasses

Contents:

12.0% calcium, 7% phosphor, 10% sodium 4% magnesium

Additives per kilogram:

1,000,000 IU Vitamin A, 135,000 IU Vitamin D (Vitamin D3), 3,000 mg Vitamin E (alpha-Tocopherol acetate), 40 mg Vitamin B1, 80 mg Vitamin B2, 40 mg Vitamin B6, 400 mg Vitamin B12, 600 mg nicotinic acid, 120 mg pantothenic acid, 20 mg Vitamin K3, 200 mg iron (Fe-(II)-carbonate), 2,000 mg copper (Cu-(II)-sulphate), 9,000 mg zinc (zinc sulphate), 4,000 mg manganese, 180 mg iodine, 60 mg selenium (sodium selenite), 45 mg cobalt

*grape seed extract contains natural antioxidants that are 18,4 times as potent as Vitamin C and 50 times as potent as Vitamin E.

Recommended dose: 80-100 g/animal/day

*Fulminant Phos 9/10 (mineral feed, dry cows), Fulminant GmbH, Stockach-Zizenhausen, Germany*

Composition:

Calcium phosphate, sodium chloride, calcium-sodium-magnesium-phosphate, magnesium oxide, yeast, sugar beet molasses

Contents:

9.0% calcium, 10% phosphor, 10% sodium 4% magnesium

Additives per kilogram:

1,000,000 IU Vitamin A, 150,000 IU Vitamin D (Vitamin D3), 2,000 mg Vitamin E (alpha-Tocopherol acetate), 30 mg Vitamin B1, 45 mg Vitamin B2, 25 mg Vitamin B6, 200 mg Vitamin B12, 150 mg nicotinic acid, 70 mg pantothenic acid, 10 mg Vitamin K3 200 mg iron (Fe-(II)-carbonate), 5 mg folic acid, 1,000 mg copper (Cu-(II)-sulphate), 8,000 mg zinc (zinc sulphate), 4,000 mg manganese, 80 mg iodine, 50 mg selenium (sodium selenite), 20 mg cobalt

Recommended dose: 120-150 g/animal/day

*Bovigold 164 (grain mix, lactating animals), RKW Süd, Regensburg, Germany*

Composition:

16.0% crude protein, 2.8% raw fat, 8.9% crude fibre, 5.8% ash, 0.70% calcium, 0.65% phosphor, 0.2% sodium, 7 MJ NEL/kg

Additives per kilogram:

1,000 IU Vitamin A, 1.000 mg Vitamin D3, 20 mg copper (Cu-(II)-sulphat
