# Supplementary material for: Alopecia in Belgian Blue crossbred calves: a case series
Source: BMC Vet Res. 2019 Nov 15;15:411. doi: 10.1186/s12917-019-2140-1 (PMC6858713; doi:10.1186/s12917-019-2140-1)
Supplement: Supplementary file 2 — Additional file 2. Results of feedstuff analysis. Percent dry matter, net energy content for lactation, percent protein, percent fibre, percent fat, percent ash, calcium, phosphorus, sodium, potassium, magnesium, iron, zinc, copper, chloride and manganese of grass silage, hay and corn pellets fed to cows at the dairy farm. [file 12917_2019_2140_MOESM2_ESM.docx]

**Additional file 2 – Results of feedstuff analysis**

| Feedstuff | % dry matter | MJ NEL/kg | % protein | % fibre | % fat | % ash | Ca g/kg | P g/kg |
| --- | --- | --- | --- | --- | --- | --- | --- | --- |
| Grass silage | 56.8 | 6.2 | 6.5 | 26.3 | 2.7 | 9.3 | 7.7 | 5.1 |
| Hay | 88.4 | 4.6 | 9.6 | 39.8 | 1.3 | 7.5 | 2.9 | 4.3 |
| Corn pellets | 88.5 | 6.6 | 15.7 | 20.8 | 1.1 | 3.3 | 1.9 | 4.1 |

| Feedstuff | Na g/kg | K g/kg | Mg g/kg | Fe mg/kg | Zn mg/kg | Cu g/kg | Cl g/kg | Mn mg/kg |
| --- | --- | --- | --- | --- | --- | --- | --- | --- |
| Grass silage | 0.2 | 36.2 | 1.9 | 121.4 | 27.7 | 9.0 | 6.1 | 75.7 |
| Hay | 0.1 | 28.8 | 1.1 | 215.7 | 27.7 | 9.0 | 6.1 | 75.7 |
| Corn pellets | 0.1 | 8.7 | 1.1 | 157.0 | 19.4 | 4.6 | 1.4 | 27.6 |
