# Supplementary material for: Alopecia in Belgian Blue crossbred calves: a case series
Source: BMC Vet Res. 2019 Nov 15;15:411. doi: 10.1186/s12917-019-2140-1 (PMC6858713; doi:10.1186/s12917-019-2140-1)
Supplement: Supplementary file 3 — Additional file 3. Summary of the computer assisted calculation of the lactating cow ration. Calculated wet weight, dry matter, net energy content for lactation, raw protein, ruminal nitrogen balance, calcium, phosphorus, magnesium and sodium content of the lactating cow ration using a computer assisted calculation program. [file 12917_2019_2140_MOESM3_ESM.docx]

**Additional file 3 – Summary of the computer assisted calculation of the lactating cow ration (MiFuBo 2001).**

| Item | Wet weight (kg) | Dry matter (kg) | NEL (MJ/ kg) | Raw protein (g) | RNB^a^ (g) | Ca (g) | P (g) | Mg (g) | Na (g) |
| --- | --- | --- | --- | --- | --- | --- | --- | --- | --- |
| Grass silage | 20 | 11.4 | 70 | 761 | -4 | 87 | 58 | 21 | 2.3 |
| Hay | 3 | 2.7 | 12 | 262 | -1 | 8 | 11 | 3 | 0.3 |
| Grain mix | 3 | 2.6 | 13 | 323 | -2 | 21 | 20 | - | 6.0 |
| Corn pellets | 2 | 1.77 | 21 | 494 | -2 | 4 | 8 | 2 | 0.2 |
| Sum | 28 | 18.47 | 116 | 1840 | -9 | 120 | 97 | 26 | 8.8 |

^a^Ruminal nitrogen balance
